# New Diagnostic Algorithm for Chagas Disease: Impact on Access to Diagnosis and Out-of-Pocket Expenditures in Colombia

**Published:** 2019-07

**Authors:** Mario J. OLIVERA, Kevin A. CHAVERRA

**Affiliations:** 1. Instituto Nacional de Salud, Grupo de Parasitología, Bogotá D.C., Colombia; 2. Pontificia Universidad Javeriana, Programme in Health Economics, Bogotá D.C., Colombia

## Dear Editor-in-Chief

Despite extensive efforts to control the disease implemented since several decades, Chagas disease remains one of the biggest public health problems in Latin America, where it is caused by the parasite *Trypanosoma cruzi* (1). Globally, more than 6 million people have Chagas disease and 7 thousand persons die from it (2). In Colombia, an estimated 437,960 persons are chronically infected with *T. cruzi*, although many people with the disease are not diagnosed (3).

Making a diagnosis of Chagas disease is not easy, a single test is not sufficiently sensitive and specific to make the diagnosis. For this reason, WHO recommends applying two or more tests that use different techniques and/or detect antibodies to different antigens, which makes it financially challenging. The conventional serological tests commonly used are ELISA, indirect hemagglutination assay (IHA) and indirect immunofluorescence assay (IFA) (3).

**Fig. 1:**
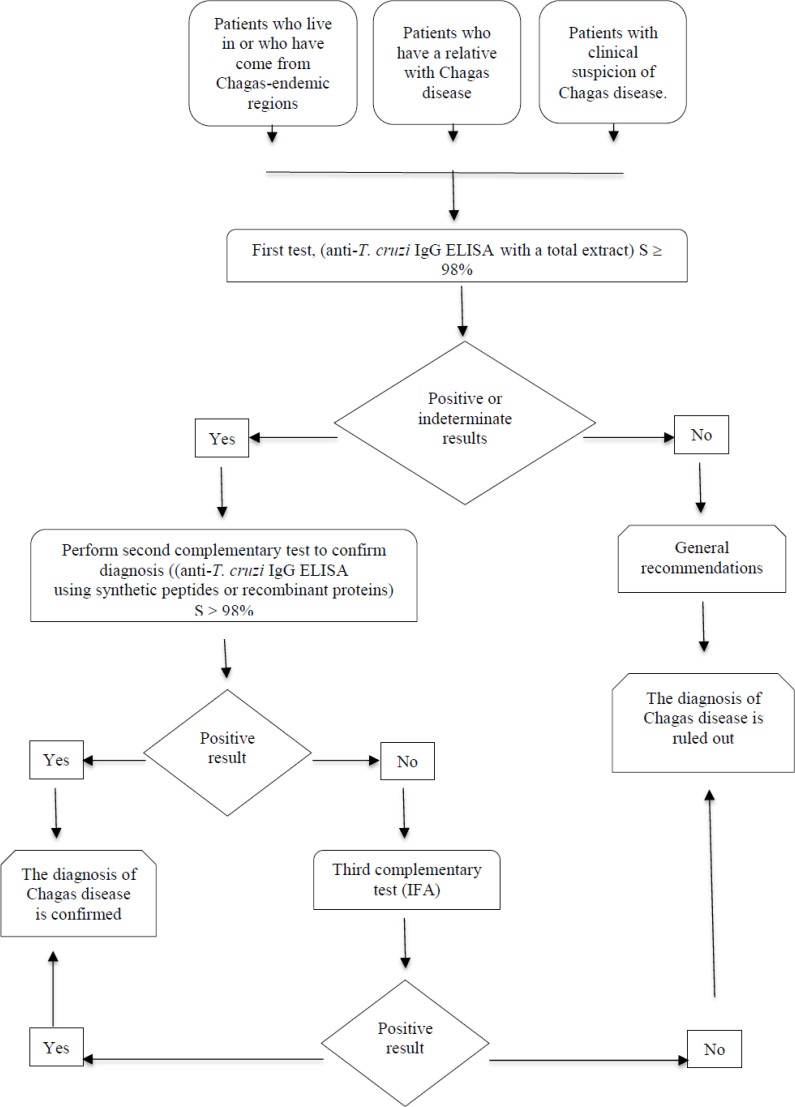
Generalized flow diagram of Chagas disease diagnosis in Colombia

People infected with *T. cruzi* must overcome different barriers to achieve diagnosis (3). To solve the logistical barriers of access to conventional serological tests, the Ministry of Health and Social Protection has generated a new diagnostic algorithm with non-conventional serological assays using recombinant *T. cruzi* proteins, which have values of sensitivity and specificity close to 100%. These non-conventional serological assays will be included in the health benefits plan and these will be used in primary care centers, close to the patients (Fig. 1).

Importantly, the Colombian health system provides financial protection against health care spending due to illness. Out-of-pocket expenditure paid by patients is only 14% of total health spending (4). This is one of the lowest proportions in Latin America. However, people infected with *T. cruzi* face financial barriers to have access to diagnosis, out-of-pocket expenses are common for accessing the confirmatory test and are exposed to financial risks due to illness (3).

The out-of-pocket payment is considered as one of the potential factors associated with catastrophic health expenditure (5). Due to such negative consequences and lack of affordability of healthcare expenditures, many people may deny demanding healthcare services, especially elective ones (6, 7). Yet, the out-of-pocket costs of patients with Chagas disease care are rarely measured.

**Fig. 2:**
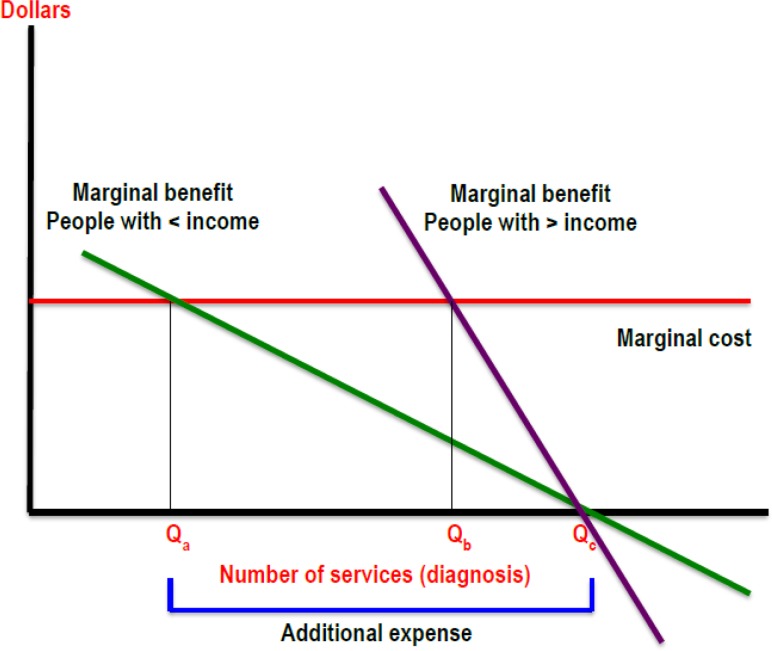
Relationship between marginal benefit and marginal cost

Meanwhile, a scenario with zero out-of-pocket spending could be the beginning of a crisis for the Colombian health system (Fig. 2). The new diagnostic algorithm could generate an excess of expenditure in relation to the social optimum, to the point at which the benefits equal marginal costs. If the cost becomes irrelevant in the decisions, the demand will go to the point where the marginal benefit is zero.

In general, patients who have to wait almost 6 months elapse between the requests of the tests and the confirmation of the disease experience higher out-of-pocket costs on diagnosis. However, the proportion of total out-of-pocket expenses for the diagnosis of Chagas disease in patients is unknown. It is crucial to recognize the need for better equity and financial protection, since high out-of-pocket expenses may influence the decisions of families to not seek adequate healthcare in order to make ends meet.
